# Characterization of CuAg Alloys with Low Ag Concentrations

**DOI:** 10.3390/ma17081823

**Published:** 2024-04-16

**Authors:** Lorenzo Mosesso, Salvatore Macis, Annalisa D’Arco, Augusto Marcelli, Andrea Notargiacomo, Marialilia Pea, Bruno Spataro, Vincenzo Stagno, Stefano Lupi

**Affiliations:** 1 Department of Physics, Sapienza University of Rome, Piazzale Aldo Moro 5, 00185 Rome, Italy; lorenzo.mosesso@uniroma1.it (L.M.); salvatore.macis@uniroma1.it (S.M.); annalisa.darco@uniroma1.it (A.D.); 2 INFN—Laboratori Nazionali di Frascati, via Enrico Fermi 54, 00044 Rome, Italy; augusto.marcelli@lnf.infn.it (A.M.); bruno.spataro@lnf.infn.it (B.S.); 3 Rome International Centre for Materials Science Superstripes, Via dei Sabelli 119A, 00185 Rome, Italy; 4 Istituto di Fotonica e Nanotecnologie, Consiglio Nazionale delle Ricerche, IFN-CNR, Via del Fosso del Cavaliere 100, 00133 Rome, Italy; andrea.notargiacomo@ifn.cnr.it (A.N.); marialilia.pea@ifn.cnr.it (M.P.); 5 Department of Earth Sciences, Sapienza University of Rome, Piazzale Aldo Moro 5, 00185 Rome, Italy; vincenzo.stagno@uniroma1.it; 6 INFN Section of Rome, Sapienza University, Piazzale Aldo Moro 5, 00185 Rome, Italy

**Keywords:** copper alloys, optical spectroscopy, scanning electron microscopy

## Abstract

Copper-based alloys designed to combine high electronic and thermal conductivities with high mechanical strength find a wide range of applications in different fields. Among the principal representatives, strongly diluted CuAg alloys are of particular interest as innovative materials for the realization of accelerating structures when the use of high-gradient fields requires increasingly high mechanical and thermal performances to overcome the limitations induced by breakdown phenomena. This work reports the production and optical characterization of CuAg crystals at low Ag concentrations, from 0.028% wt to 0.1% wt, which guarantee solid solution hardening while preserving the exceptional conductivity of Cu. By means of Fourier Transform Infrared (FTIR) micro-spectroscopy experiments, the low-energy electrodynamics of the alloys are compared with that of pure Cu, highlighting the complete indistinguishability in terms of electronic transport for such low concentrations. The optical data are further supported by Raman micro-spectroscopy and SEM microscopy analyses, allowing the demonstration of the full homogeneity and complete solubility of solid Ag in copper at those concentrations. Together with the solid solution hardening deriving from the alloying process, these results support the advantage of strongly diluted CuAg alloys over conventional materials for their application in particle accelerators.

## 1. Introduction

Alloying is typically used in metals to improve several fundamental properties, such as mechanical strength or electrical conductivity. Although these features are easy to enhance individually, their combined improvement has always been particularly challenging. Solid solution hardening, which consists of limiting the dynamics of lattice defects by the strain fields introduced by impurity substitutions, is the natural cause of material strengthening for increasing impurity concentrations [1,2]. Correspondingly, the introduction of these impurities in the lattice leads to an increase in scattering sources that limit the electronic transport, increasing the resistivity of the material. The competition between the two processes [3] severely reduces the number of alloys that simultaneously exhibit high mechanical strength and high conductivity. However, there are some notable exceptions that share both properties, the main representative of which is CuAg. This eutectic alloy exhibits a great variety of phases and microstructures that, combined with appropriate heat treatments, affect the material’s macroscopic characteristics, offering a great variety of applications by displaying an outstanding combination of strength and thermal/electrical conductivity [4,5,6,7,8,9,10].

Depending on the Ag concentration in CuAg, a wide range of applications in many different fields can be considered. For concentrations above 6%wt-Ag, the alloy is above the maximum solubility threshold of Ag in Cu so that at room temperature in equilibrium conditions, the Cu matrix phase can accommodate second (Ag) phase domains. The microstructures characterizing the system consist mainly of primary Cu dendrites surrounded by mixed eutectic regions [6,11]. In these alloys, heat treatments and their effect on the microstructures become crucial for their final characterization. Mechanical strength, while being scarcely affected by aging, depends strongly on work-hardening processes, such as cold drawing, which allows the alloy to obtain additional hardening through the realization of filamentary structures [12,13]. The conductivity, on the other hand, is reduced overall by the increase of Ag in the solid solution. For this reason, CuAg with concentrations above 6%wt is typically used in applications where high mechanical strengths are required at the expense of small reductions in electrical conductivity (tensile strength above 1 GPa with conductivities up to 80%IACS have been reached [4]). A notable example of this application is the realization of wire windings for pulsed high-field magnets [13].

Below 6%wt-Ag, the alloy is commonly cast in its metastable single solid solution phase, in which eventual Ag-matrix particles are introduced through precipitation hardening. The absence of a complex network of microstructures limits the increase in mechanical strength through work hardening, making the aging processes more effective [11]. As a result, low-concentration alloys or strongly diluted alloys are commonly used in applications where it is required to preserve the incredible conductivity properties of Cu while also ensuring a non-negligible increase in material strength. A very important example of such an application is for radiofrequency (RF) cavities in particle accelerators. Recently, great efforts have been made to operate the cavities at high accelerating gradients above 100 MV/m, which is the current threshold imposed by RF breakdown for conventional Cu-based structures [14,15]. Although the phenomenology of RF breakdown is still not completely understood, some precursor effects have been confirmed. For example, the breakdown rate can be triggered by RF pulse heating phenomena [16,17,18,19], which induce thermal stresses that increase the mobility of the dislocations toward the surface regions, causing the formation of protrusions that, by increasing the local fields, activate dielectric breakdowns [20]. Therefore, the need for materials such as diluted CuAg alloys becomes immediately apparent. Providing high conductivity and mechanical strength, they guarantee, on the one hand, the reduction of thermal dissipation underlying pulse heating and, on the other, the localization of defects due to solution hardening, allowing a reduction in the breakdown rate and an overall improvement in cavity performances [14,15].

The aim of this study, oriented to the latter applications, is to explore low-concentration or strongly diluted CuAg alloys (up to 0.1% wt. of Ag) prepared via the Vacuum Induction Melting (VIM) technique to maintain copper’s conductivity while enhancing material strength. Additionally, it aims to characterize the optical properties of Cu and CuAg alloys, emphasizing applications in RF cavities and investigating complete Ag solubility in the samples. To this end, Fourier Transform Infrared Spectroscopy (FTIR) experiments were carried out to obtain quantitative information on the low-energy electrodynamics of the different samples, while spectrophotometry experiments allowed to access their electronic response at higher frequencies up to the ultraviolet (UV). These electromagnetic measurements were further supported by scanning electron microscopy (SEM) and Raman micro-spectroscopy investigation for surface (textural) and compositional characterization. We experimentally demonstrate that for the range of Ag concentrations up to a maximum of Ag-0.1% wt, the electromagnetic response of CuAg alloys is practically indistinguishable from that of bare Cu so that the systems behave identically in terms of low-energy electrodynamics. This feature provides the basic requirement for the application of such diluted alloys in RF cavities and highlights the advantages of these materials with respect to conventional copper. Furthermore, the presence of Ag in the solution was not detected by any of the proposed techniques, demonstrating the complete solid solubility of Ag in our samples.

## 2. Materials and Methods

### 2.1. Sample Description and Growth Process

Silver-bearing oxygen-free copper alloys (CuAg OFS) with different low Ag concentrations were investigated and compared with a pure metallic oxygen-free copper sample (C10100). The available concentrations, expressed in %wt-Ag, are 0.028, 0.06 and 0.1%wt. 

The samples were produced by RINA Company (Genoa, Italy) with a Vacuum Induction Melting (VIM) plant, using an ad hoc procedure designed for the proposed alloys. The VIM melting process consists of the fusion of the required metal charge into a refractory crucible, bringing it from room temperature up to the liquidus temperature, exploiting the Joule effect induced by eddy currents generated by a water-cooled induction coil surrounding the crucible. The melting chamber is equipped with a vacuum system that allows the reduction of the atmospheric pressure down to a target working point (usually 0.01 mbar or less). The cold metal charge, loaded within the refractory crucible at the start of the process, is already inclusive of the alloying element (Ag). However, it is possible to introduce the alloying compound later in the molten by means of a filler system placed on the top of the furnace. The first step in the process is to reduce the atmospheric pressure within the crucible using the vacuum pumps. After that, the power group of the plant is switched on to heat the metal up to the melting temperature. It is important for the metallic charge to increase its temperature under vacuum conditions in order to reduce the formation of surface oxides during heating. Furthermore, the melting process is characterized by two competing effects: in one case, to reduce the percentage of some low-boiling elements, it is necessary to maintain the vacuum for a very long time. In the other case, the extended duration of the vacuum/melting process can cause pollution to the alloy due to the interaction with the crucible. Therefore, the best compromise between time duration, vacuum level and temperature was experimentally selected for the sample growth. The various stages of the process, with the corresponding thermodynamic conditions, are reported in Table 1.

Several procedures have been tested to achieve the desired quality for the final product. In fact, different configurations and materials for the crucible have been considered, together with different atmospheric and pressure conditions. Moreover, different casting and solidification processes have been studied in order to obtain homogeneous alloys. After several tests and chemical analyses, the best choice was to melt Cu and Ag together within a graphite crucible, followed by casting in a ceramic shell in an inert atmosphere of argon. Possible alternative solidification processes have been excluded as they showed uneven chemical analyses along the longitudinal section of the ingot produced, with variations in the Ag content. This phenomenon was attributed to Ag segregation phenomena given by low solidification speeds. It was therefore decided to use ceramic shell casting in order to have a faster solidification, avoiding the predisposition to segregation phenomena and obtaining a more homogeneous material.

The final samples, obtained by electro-erosion machining off the primary ingot, consist of cylindrical shapes with a diameter of 5 mm and thickness between 1- and 2-mm. Different surface processing was required for each of the available samples to ensure the best possible accuracy on optical measurements. Hence, all samples were exposed to a mechanical polishing process to achieve surface smoothness within an accuracy of 100 nm and to reduce the residual scratch density on the surface over a spatial scale larger than the spatial resolution of our optical micro-spectroscopy probe techniques.

### 2.2. Methods

Light, used as a probe, is a powerful tool to gain access to the physical detail of a generic target in condensed matter. In fact, by measuring macroscopic quantities, such as absorption, reflectance or transmittance, it is possible to achieve a deep connection with the microscopic description of the system through the introduction of analytical models of varying sophistication. Here to optically characterize conventional metals such as Cu and its CuAg alloys, various optical spectroscopy techniques were used to cover a wide frequency range from far infrared (IR) to UV. Optically, all samples can be considered as infinite slabs, as the penetration length of the electromagnetic radiation in Cu and Ag is much smaller than the sample thickness (1–2 mm). This, together with the high metallicity of Cu, demands reflectance setups in all the proposed experiments since sample transmittance is zero up to the Cu plasma edge $\omega ∼20,000\;cm^{-1}$ ($8000\;cm^{-1}∼1\;eV$). Low-frequency reflectance measurements were collected using a Vertex V70 FTIR (Bruker, Billerica, MA, USA) broadband interferometer coupled with a Bruker Hyperion 2000 infrared microscope. Different combinations of sources (globar or halogen lamp), beamsplitters (Si or quartz) and detectors (nitrogen-cooled MCT) were used. Reflectance measurements were collected at near-normal incidence, requiring complete flatness for the surface of the samples in order to avoid diffusive effects limiting the sensitivity of the measurements. For this purpose, the microscope was equipped with a mobile sample holder, providing pitch and roll adjustment within mrad accuracy and ensuring maximization of the reflected signal.

All reflectance data were collected using a thin gold layer as an optical reference. The 100–150 nm Au films were evaporated on half of the surface of each sample using an evaporation chamber with a residual pressure of at least $10^{-7}\;mbar$ and a quartz crystal microbalance to monitor the film deposition. Due to the Au films deposited on the surface, it was possible to have a mirror reference that reproduces as close as possible the surface characteristics of all Cu and CuAg samples, thus minimizing sample-related experimental errors.

Reflectance measurements from NIR to UV were carried out using the JASCO v770 spectrophotometer (JASCO, Tokyo, Japan). Before making any optical measurements, all samples were treated with antioxidant products and then cleaned in acetone and ethanol baths, removing the surface oxidative layer.

Once the reflectance of all samples has been measured in the whole frequency range of interest, the dielectric function $\hat{\varepsilon }(\omega )$ of each crystal could be accessed by fitting experimental data with the analytical Drude–Lorentz model [21]:

(1) $$\hat{\varepsilon }\left(\omega \right)={\hat{\varepsilon }}_{\infty }+{\hat{\varepsilon }}_{Drude}\left(\omega \right)+{\hat{\varepsilon }}_{Lorentz}^{elect.}\left(\omega \right)={\hat{\varepsilon }}_{\infty }-\frac{\omega_{p}^{2}}{\omega^{2}+i\gamma \omega }+\sum_{i}\frac{\omega_{pi}^{2}}{\omega_{0i}^{2}-\omega^{2}-i\gamma_{i}\omega }.$$

here $\omega_{0i}$, $\omega_{pi}$ and $\gamma_{i}$, which are the resonant frequency, the plasma frequency and the scattering rate associated with the *i*-th excitation, respectively, are the free parameters of the fitting process, in which R is calculated analytically from the imaginary and real part of the dielectric function via the Fresnel formula:

(2) $$R={\left|\frac{1-\sqrt{\hat{\varepsilon }}}{1+\sqrt{\hat{\varepsilon }}}\right|}^{2}.$$

Finally, the optical conductivity $\hat{\sigma }(\omega )$ is immediately determined by $\hat{\varepsilon }\left(\omega \right)=\frac{4\pi i}{\omega }\hat{\sigma }(\omega )$ once the fit parameters are fixed. Optical data are fitted using *RefFit* software (https://reffit.ch/, accessed on 11 April 2024) [22].

To support optical spectroscopy experiments, Raman micro-spectroscopy and SEM microscopy measurements were carried out to highlight any compositional differences between the alloys at different concentrations and to give surface characterization in order to confirm the quality of the optical measurements, which are sensitive to surface properties within the skin depth. The JASCO NRS-5100 laser Raman spectrometer was used for Raman measurements. It is equipped with a spectrograph in a Czerny-Turner configuration that allows spectra to be collected in the Raman shift range of 50–8000 $cm^{-1}$ with a resolution of $3\;cm^{-1}$. For our purpose, the spectral region from 200 to 1000 $cm^{-1}$ was sufficient to observe the main features of the samples. A rejection filter (edge filter) was used to remove the Rayleigh elastic contribution, while a 4-stage Peltier-cooled CCD array was used as a detector. Of the two possible operational wavelengths of the instrument, 785 nm and 532 nm, respectively, only the red one is used in this work. Raman spectroscopy is used here to identify Cu and Ag oxides on sample surfaces to discriminate Cu from its CuAg alloys.

SEM measurements were carried out using a FEI Quanta 400 (Thermo Fisher Scientific, Waltham, MA, USA) Microscope coupled with an EDAX Genesis Microanalysis System. It is equipped with an Everhart Thornley detector for secondary electrons and also with a Quad Solid State back-scattered electrons detector to detect contrasts between areas with different chemical composition. Finally, it enables the semiquantitative analyses of the elements making up the samples by means of energy dispersive spectroscopy (EDS).

## 3. Results and Discussion

### 3.1. Optical Spectroscopy Results

**Figure 1:**
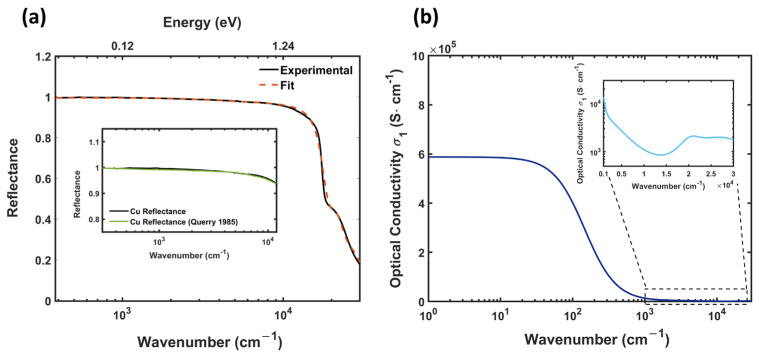
Reflectance and optical conductivity for pure copper—(**a**) Cu reflectance defined in the spectral range from MIR to UV (black line) together with the Drude–Lorentz optical fit (red dashed line) obtained by means of Equations (1) and (2). In the inset, the experimental reflectance (black line) is compared with the literature data [23] (green line) in the IR spectral range, where R is almost constant with values above 0.99. (**b**) Real part $\sigma_{1}(\omega )$ of the optical conductivity $\hat{\sigma }(\omega )$ extracted from the Drude–Lorentz fit with the high-frequency region magnified to highlight inter-band contributions.

The starting point of this study is an accurate optical characterization of metallic copper. Figure 1a shows the experimental reflectance data of Cu over the whole frequency range from IR to UV. As expected from the optical response of a conventional metal, reflectance shows an approximately constant behavior at frequencies lower than the plasma edge, with values R>0.996 in the FIR region. In the inset of Figure 1a, our final measured result (black line) is compared with the experimental data reported in Querry et al. [23] (green line), showing an excellent matching. 

At higher frequencies, Cu exhibits a plasma edge at $\omega_{edge}∼17,600\;cm^{-1}$, beyond which the reflectance R decreases significantly from R>0.95 to R<0.45. The microscopic interpretation of this behavior can be explained in terms of the semiclassical Drude–Lorentz model, which allowed the experimental reflectance data to be fitted (red dashed line in Figure 1a) by the combination of Equations (1) and (2), as reported in Section 2.2. The low-energy response, associated with the electronic transport, is completely dominated by the Drude contribution, which exhibits a plasma frequency value [24] $\omega_{p}∼72,000\;cm^{-1}$ well above the plasma edge $\omega_{e}$ and a scattering rate value γ compatible with the high conductivity of the material. On the other hand, the inter-band electronic transitions, described by Drude–Lorentz oscillators centered around 2.4 eV and 5 eV and mainly associated with d electron transition to states above the Fermi energy [25,26], are responsible for the absorption onset associated with the strong decrease in reflectance around the plasma edge. Once the fit parameters are fixed, the Drude–Lorentz model provides the determination of the linear response functions of the system, including the complex optical conductivity $\hat{\sigma }(\omega )$, whose real part $\sigma_{1}(\omega )$ is shown in Figure 1b. The entire frequency response, as expected, is completely dominated by the Drude term, which yields very high conductivity values at low frequencies. Given ω→0, it is possible to extrapolate the very important value $\sigma_{DC}=5.86\times 10^{5}\;S\;cm^{-1}$ from $\sigma_{1}(\omega ),$ which confirms the expected 101%IACS DC conductivity for the sample.

Once copper has been fully characterized, the next step is to examine the optical response of all the available diluted metal alloys. Particular attention will be paid to samples with concentrations of 0.1 and 0.06%wt-Ag as they provide all the necessary conclusions we need. The optical and electronic properties of noble metal-noble metal alloys are already studied in the literature [26,27,28], both for large concentrations of silver [28] and for dilute alloys [26]. However, all these works propose the characterization of thin film samples that are thermally treated to host a complete metastable CuAg phase. Metastability is hard to achieve since CuAg alloys have very low solubility limits in their phase diagram, already lower than 0.2%wt at 200° C [29]. The choice of thin films is obvious since they intrinsically provide polished surfaces for appropriately chosen thicknesses, while metastability is necessary to avoid inhomogeneities introduced by possible precipitations. In fact, optical properties are critically affected by the presence of microstructures and segregations within the material. In the present case, the analysis of the optical properties is carried out on bulk systems exhibiting a single solution phase (as will be demonstrated below with the microscopy results) with Ag diluted uniformly to incredibly low concentrations. Since the measurements are not performed on thin films, the limitation imposed on the optical measurements is dictated by the quality of the polishing process, which guarantees almost mirror-like surface regions with the presence of some residual scratches distributed on spatial scales greater than the spatial resolution of the optical micro-spectroscopy technique.

**Figure 2:**
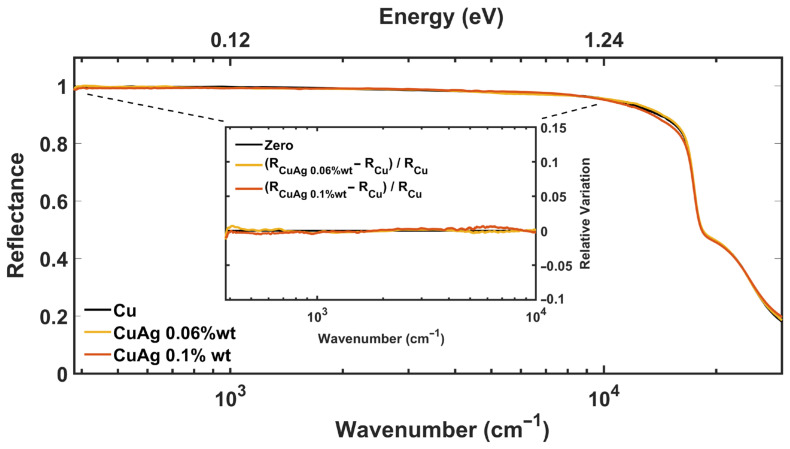
**Differential Reflectance Method for CuAg alloys**—CuAg reflectance for 0.1%wt and 0.06%wt-Ag concentrations are presented in the whole spectral range from FIR ($300\;cm^{-1}$) to UV ($30,000\;cm^{-1}$) together with Cu reflectance. The sample with 0.028%wt-Ag is not reported as it does not add any further information. The relative variation of Reflectance for different Ag concentrations is reported in the inset as a function of the wavenumber in the IR spectral range.

Figure 2 shows the absolute reflectance of CuAg 0.1%wt, and CuAg 0.06%wt compared with Cu reflectance in the whole examined spectral range. As the expected variations on the optical measurements are very small due to the low Ag concentrations, the analysis of the optical response is supported by the differential reflectance method, in which the relative change of $R_{CuAg}$ is measured with respect to the reference reflectance $R_{Cu}$ by $(R_{CuAg}-R_{Cu})/R_{Cu}$. In the IR spectral range, both concentrations show a very small variation with respect to the bare Cu, highlighting the same optical behavior within a 0.3% error. The slight fluctuations visible in the lowest part of the spectrum of Figure 2 are related to the detection noise. This result clearly indicates that the electromagnetic properties of alloys within the studied concentrations are indistinguishable from Cu. That is, in terms of low-energy optical properties, copper alloys behave exactly as pure copper. These results confirm the considerations in the Handbook of Copper and Copper Alloys [29], where all the proposed concentrations exhibit exactly the same %IACS and an identical nominal DC resistivity value as Cu.

As a final remark, it is important to stress that the spatial resolution and the local information provided by this spectroscopic technique do not limit the validity of the proposed results on a macroscopic scale. The uniformity of the Ag solution, which will be demonstrated with the SEM and Raman results in the next sections, guarantees the same Ag atomic density in every surface region of the sample with expected small fluctuations so that the equivalence of the optical response should not be ascribed to the sampling of possible Ag-free spatial regions.

### 3.2. SEM Microscopy Results

Reflectance measurements performed on metallic samples involve the penetration of radiation within a characteristic distance (penetration depth), which typically depends on frequency. This distance usually extends over a thin layer below the surface, ensuring the probe’s sensitivity to surface properties such as oxidation. In addition, all optical measurements are affected by the presence of microstructures, precipitations or compositional impurities that can appear over the surface, substantially modifying the local optical response of the system. For this reason, microscopy experiments can be used as a relevant support to optical spectroscopy measurements. This support is even more relevant if, in combination with the topographic characterization of the sample surface, such microscopy techniques allow for different quantitative analyses, such as the chemical identification of the elements within the material. In this respect, SEM is the ideal tool to study noble metals and their alloys. Through the detection of secondary electrons (SE) and back-scattered electrons (BSE), it is possible to access distinct information on the same surface under investigation with different surface depth resolutions. The SEs, which are much less energetic than the primary beam electrons, come from the very first layers of the sample, providing the topographic representation of its surface. BSEs, on the other hand, may come from inner layers revealing the different chemical composition within the material. The former allows for the distinction of grains or impurity inclusions within the specimen, while the latter discriminates the presence of microstructures or precipitations resulting from the heat treatment process used for the synthesis of the alloys. 

**Figure 3:**
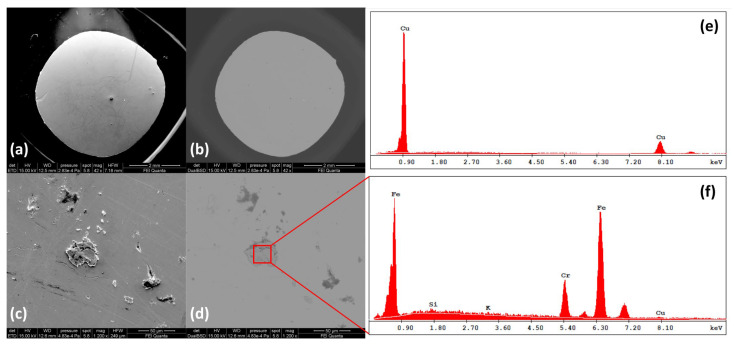
Analysis by scanning electron microscopy—(**a**,**b**) Images of the CuAg 0.028%wt sample surface obtained with secondary (SE) and back-scattered electrons (BSE), respectively. By color contrast in (**b**), the complete solubility of the alloy and the absence of Ag precipitations is immediately apparent. Similar images are found for the other concentrations. (**c**,**d**) Details of impurity grains randomly distributed over the sample surface. The color contrast in (**d**), highlighted by the red box, indicates a different chemical composition. Quantitative analysis of the chemical elements constituting the impurity is provided by EDS measurements. (**f**) indicates the composition of the grain, while (**e**) shows the composition of the CuAg sample at an arbitrary point on the surface.

Figure 3a,b shows the surface of the CuAg 0.028%wt sample measured with both the electron probes. Similar images with similar characteristics were collected for all concentrations. These images are particularly relevant because they show an essential feature of the sample, namely its surface homogeneity. From the color contrast in Figure 3b, it is immediately clear that the alloys at the concentrations of interest exhibit complete solubility of silver in Cu and the complete absence of microstructure or exsolution resulting from chemical unmixing owing to the thermal evolution of the alloy. The only evidence of compositional variation on the surface is related to the presence of minimal impurities related to the growth process, all of which are randomly distributed over the surface. An example of impurities is presented in Figure 3c,d, where chemical contrast in BSE analyses is highlighted by the red box. Figure 3f shows the EDS spectrum for the compositional characterization of the impurity of Figure 3c. The peaks at 0.7 and 6.39 keV identify the transitions from the L and K shells of Fe, while the peak at 5.4 keV identifies the presence of Cr. It is interesting to note that the produced samples can be characterized by different impurities with different compositional natures depending on the growth environment of the distinct batches.

Figure 3e shows the EDS spectrum taken at a generic point on the surface of the 0.028%wt CuAg sample. Only the Cu peaks associated with the L and K transitions appear, while no trace of silver, whose main peaks are expected in the range between 2.70 and 3.60 keV, is detected. This is similarly true for the higher concentrations, where the Ag peaks are always covered by the background signal. Similar spectra are also obtained by performing EDS mappings over extended regions ($∼10\times 15\;\mu m^{2}$) of the sample surface, clearly demonstrating that the Ag concentration is too low to be detected by this spectroscopic technique. Thus, the BSE images and the EDS spectra highlight the absence of inhomogeneous regions with high Ag concentration, and the lack of precipitation and Ag-based microstructures demonstrates once again the realization of the metastable state with a single solid solution phase for the dilute CuAg alloys.

Despite the surface sensitivity of the experimental methods proposed in this work, the realization of the uniform solid solution measured at the surface of each sample can be extended to the bulk of the metal alloys. In fact, the investigations with SEM, EDS and optical spectroscopy techniques (both FTIR and Raman) were carried out on the top and bottom surfaces of several cylindrical samples obtained by electro-erosion machining from the same primary ingot, showing the same homogeneity and the same surface properties on every cross-section of the primary specimen.

### 3.3. Raman Spectroscopy Results

To confirm the results obtained from SEM microscopy experiments, we introduce here the Raman spectroscopy results carried out on Cu and CuAg samples to compare the spectrum of the alloys with that of pure Cu, with the aim of identifying possible differences to be attributed to the addition of larger Ag concentrations. It is important to emphasize that in the case of Cu and its alloys, Raman measurements mainly involve the identification of oxides on the surface layers since the metallic response alone does not lead to the observation of any vibrational peaks. For this reason, possible discrimination between Cu and CuAg alloys is based on the identification of peaks assigned to Ag oxides. 

**Figure 4:**
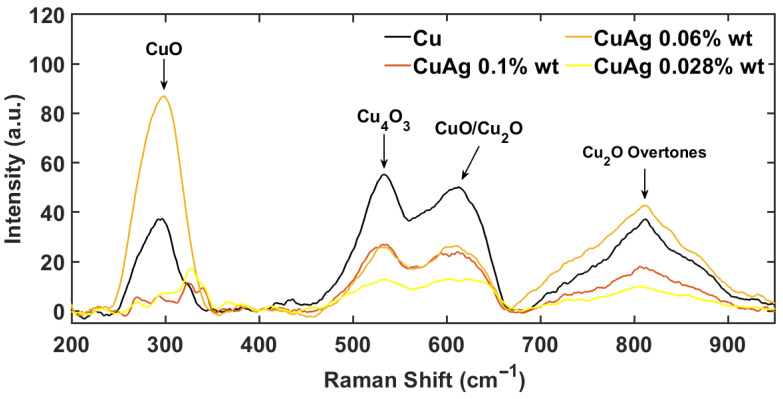
Raman micro-spectroscopy on Cu and CuAg alloys—Raman spectra collected in the Raman shift interval from $200\;cm^{-1}$ to $1000\;cm^{-1}$ for pure copper and for every Ag concentration. Black arrows indicate the main oxidation peaks in the spectra.

Figure 4 shows the Raman spectra of all samples in the frequency range from $200\;cm^{-1}$ to $1000\;cm^{-1}$, where the main spectral features of the different materials can be identified. A wider frequency range does not add any further physical information. Due to the local nature of the spectroscopic technique, different positions on the surface have been measured, confirming the same Raman peaks in the selected frequency range. All the spectral features in the CuAg samples, identified by black arrows in Figure 4, are also visible in pure Cu and are attributed to the family of copper oxides CuO, Cu_2_O and Cu_4_O_3_ [30,31,32,33,34,35,36]. So, no trace of silver oxide is detected due to the low concentration of Ag in the alloys. The different intensities of the peaks indicate different oxidation levels of the various samples, which had been subjected to different environmental conditions and treatments before the measurements. The spectral feature around $300\;cm^{-1}$ is associated with the superposition of the two symmetric under-inversion and non-degenerate vibrational modes of CuO, namely $A_{g}$ and $B_{g}$ [31,34,35,36]. The position of these modes is strongly influenced by the nature of the oxidation and by the sample properties so that any residual signals in the two samples at 0.1%wt and 0.028%wt can also be assigned to these lattice vibrations. Moreover, the last of the three active Raman modes of CuO is resonant at higher frequencies around $∼620\;cm^{-1}$ [34,36], where another spectral feature is experimentally observed. In the vicinity of this spectral region, another Raman peak is visible at $∼530\;cm^{-1}$, which is attributed to the non-degenerate vibrational mode $A_{1g}$ of Cu_4_O_3_ [33,34]. Both peaks measured in the range between $400\;cm^{-1}$ and $800\;cm^{-1}$ also contain vibrational contributions from Cu_2_O, which introduces several vibrational modes into the Raman spectrum that are not necessarily Raman active, such as the three-fold $T_{2g}$ (Raman Active) and the $3T_{1u}$ (IR active) associated with both longitudinal optical phonons (LO) and transverse optical modes (TO) [32,34]. The possibility to experimentally observe non-Raman active modes is due to the fact that Cu_2_O oxidation layers are strongly affected by intrinsic defects that locally reduce the symmetry of the system inducing the activation of forbidden modes. This is confirmed by the observation of a vibrational peak at $∼810\;cm^{-1}$, which is attributed to an overtone obtained by the combination of two Raman inactive $T_{1u}(LO)$ modes [32].

Additional peaks, which could possibly be ascribed to surface formations of Ag oxides, are not visible within the experimental resolution of the technique, confirming again the absence of precipitation or microstructures with high Ag concentration.

## 4. Conclusions

The present study has been developed within a scientific framework aimed at improving the performance of accelerating devices in particle accelerators by enhancing the key material of such structures, i.e., Cu, by realizing Cu-based alloys that simultaneously exhibit high mechanical strength and high thermal and electrical conductivity properties necessary to overcome the current threshold imposed by breakdown phenomena. Among the candidates showing these properties combined together, CuAg alloys are very promising representatives, especially when realized as uniform solid solutions with low Ag concentrations, where solution hardening is achieved without affecting the strong conductivity of Cu. Here, the realization of dilute CuAg alloys via Vacuum Induction Melting has been proposed. Different CuAg samples prepared with low Ag concentrations defined in the range 0.028%wt to 0.1%wt have been measured together with pure Cu using optical spectroscopy methods sampling a wide frequency range up to UV. Several treatments were carried out on the surface of each sample to optimize sensitivity to small changes in Ag. In particular, mechanical polishing was applied to increase surface smoothness to an accuracy within 100 nm and to remove scratches densely distributed over the surface. Antioxidant products, together with acetone and ethanol, were used to remove surface oxidative layers to the best of technical possibility, and finally, Au films were grown over the samples’ surface to reproduce superficial imperfections. The frequency-dependent metal response experimentally demonstrates the equivalence of the different samples in terms of low-energy electrodynamics, indicating the independence of the electromagnetic response from the Ag addition. This equivalence is also very promising as it highlights the apparent advantages of these diluted alloys with respect to pure copper for application in particle accelerators [37]. 

To support optical spectroscopy measurements, SEM microscopy and Raman micro-spectroscopy experiments were also carried out, allowing the surface of the samples to be characterized both in structural and chemical detail. The results of these experiments showed that the composition of each alloy was essentially uniform and free of precipitation or microstructures with high Ag concentration, demonstrating the presence of a metastable state with a single solid solution phase.

## Figures and Tables

**Table 1:** Operating conditions in the VIM growth process of the investigated CuAg alloys.

Operation	T (in °C)	Power (kW)	Pressure (mbar)	Notes
Power on		32	$10^{-2}$	Power on in vacuum to avoid oxidation
Melting	Above liquidus line	7	$10^{-2}$	
Casting	1140	*Off*	100	Top pouring into ceramic shells (investment casting) in argon atmosphere

## Data Availability

Data are contained within the article.
